# Testing front-of-package warnings to discourage red meat consumption: a randomized experiment with US meat consumers

**DOI:** 10.1186/s12966-021-01178-9

**Published:** 2021-09-08

**Authors:** Lindsey Smith Taillie, Christina Chauvenet, Anna H. Grummon, Marissa G. Hall, Wilma Waterlander, Carmen E. Prestemon, Lindsay M. Jaacks

**Affiliations:** 1 Carolina Population Center, University of North Carolina, 123 West Franklin Street, Chapel Hill, NC 27516 USA; 2 Department of Nutrition, Gillings School of Global Public Health, University of North Carolina, Chapel Hill, NC 27599 USA; 3 Health Promotion, Education, and Behavior, University of South Carolina Arnold School of Public Health, 915 Greene Street , Columbia, SC 29208 USA; 4 Center for Population and Development Studies, Harvard TH Chan School of Public Health, Cambridge, MA 02138 USA; 5 Department of Population Medicine, Harvard Medical School, Harvard Pilgrim Health Care Institute, Boston, MA 02215 USA; 6 Department of Health Behavior, Gillings School of Global Public Health, University of North Carolina, Chapel Hill, NC 27599 USA; 7 Lineberger Comprehensive Cancer Center, University of North Carolina, Chapel Hill, NC 27599 USA; 8 Department of Public and Occupational Health, Amsterdam UMC, University of Amsterdam, Amsterdam Public Health Research Institute, Meibergdreef 9, 1105 AZ Amsterdam, the Netherlands; 9 Global Academy of Agriculture and Food Security, The University of Edinburgh, Edinburgh, UK

**Keywords:** Sustainability, Food policy, Carbon footprint, Plant-based diets, Consumer behavior, Front-of-package labels, Food labeling

## Abstract

**Background:**

Reducing red meat is a strategy to improve public health and mitigate climate change in the United States and other high-income countries. Policies requiring warnings on the front of red meat packages are a promising intervention to shift consumers towards healthier and more sustainable food choices. We aimed to explore participants’ reactions to health and environmental warning messages about red meat.

**Methods:**

Between June and July 2020, we recruited a national convenience sample of US red meat consumers (*n* = 1,235; mean age 44 years) for an online survey. Participants were randomly assigned to one of four label conditions: no-label control, health warning, environment warning, and combined health and environment warning (both warnings shown side-by-side). Participants viewed three types of burritos (red meat [steak], chicken, and vegetarian) and selected their preferred item (primary outcome), the item they perceived to be most damaging to health, and the item they perceived to be most damaging to the environment (secondary outcomes). Participants then viewed their assigned warning on a series of other red meat products (no-label control participants were randomly re-assigned to one of the warning conditions) and rated the warnings on perceived message effectiveness, believability, negative emotions, perceived risk, attention, and learning something new. Finally, participants reported their intentions to reduce red meat consumption.

**Results:**

There were no significant differences in selection of the steak burrito between label conditions or in selection of the item most damaging to the environment. Those exposed to the health warning were more likely to select the steak burrito as most damaging to health compared to those exposed to other label conditions (health 73 %, combined 64 %, environment 60 %, no-label control 63 %, *p* < 0.05). The combined and health warnings elicited higher perceived message effectiveness ratings than the environment warning (combined mean 2.91, health 2.84, environment 2.61, *p* < 0.05).

**Conclusions:**

Warnings did not have a significant effect on item preference in the choice experiment. However, combined and health warnings performed better than the environment warning across a variety of warning label reaction measures. More research will be needed to understand whether warnings elicit behavioral change in real-world environments.

**Trial registration:**

Analyses and hypotheses were preregistered on https://aspredicted.org/ph7mb.pdf on 23 June 2020.

**Supplementary Information:**

The online version contains supplementary material available at 10.1186/s12966-021-01178-9.

## Background

Red meat consumption is damaging to human and planetary health. The International Agency for Research on Cancer (IARC) recognizes processed red meat as a Group 1 carcinogen (“carcinogenic to humans”) and unprocessed red meat as a Group 2 A carcinogen (“probably carcinogenic to humans”) [1]. This relationship is strongest for colon and rectal cancer [1, 2]. Red meat production also contributes to climate change [3, 4]. Greenhouse gas emissions from beef production are more than 50 times that of fruit, vegetable, and grain production and more than 7 times than that of chicken [5].

On average, US adults consume 284 g of unprocessed red meat per week [6], nearly three times the level recommended by experts to promote human and planetary health [7]. Public policies offer potential to reduce red meat consumption at the population level because they impact millions of people at once [8]. One such policy is the requirement of warnings on the front of red meat packages, a policy that has been used to reduce consumption of other harmful products. For example, the United States has long had health warnings on tobacco products, which have been effective at reducing sales and use [9, 10], and Canada recently required health warnings on cannabis products [11]. Health warnings have been required for sugar-sweetened beverage advertisements in San Francisco and seven states have proposed legislation requiring health warnings on sugar-sweetened beverages [12]. Internationally, since 2016, seven countries (most in Latin America) have implemented or are implementing front-of-package nutrient warnings on foods and beverages high in sugar, sodium, saturated fat, or other nutrients of concern [13]. Thus, a policy that requires warnings on the front of red meat packages is a potential option to reduce red meat consumption.

However, little is known about the effect of warnings on red meat or consumer perceptions and choices. This is particularly relevant, considering that most previous labeling efforts have focused on warning about the health effects of a product, whereas for meat, labels are motivated by concerns about both health and environmental harms. In the environmental context, most research on labeling red meat has focused on carbon labels, and several countries and private companies have voluntary carbon labeling on food products [14, 15]. In the health context, most research on health warnings has focused on sugar-sweetened beverages, with a recent meta-analysis finding that health-based warnings (e.g., “This product contributes to obesity”) were perceived as effective by consumers and that such warnings lowered perceptions of healthfulness, increased perceptions of disease likelihood, and decreased intentions to purchase [16]. Other studies have shown that health warnings on sugar-sweetened beverages reduce consumers’ purchases of these products [17]. Tobacco research suggests that health warnings help people quit smoking [18, 19] as well as change consumer perceptions and emotions predictive of longer-term behavior change [9, 10, 20]. However, there is a dearth of evidence on similar warnings on red meat. It is currently unclear whether environmental or health warnings on red meat products would be perceived as effective, alter consumers’ perceptions about the health risks of consuming red meat, or change their intentions to reduce red meat consumption, key steps on the pathway to behavior change [21].

The objective of this study was to experimentally test health and environmental warnings about red meat among US red meat consumers as a first step to informing potential labeling or other communications policies related to red meat. Specifically, we compared the message effectiveness of health, environment, or combined health and environment warnings. Our primary outcome was selection of a red meat item in a choice experiment. Secondary outcomes included perceived message effectiveness, risk perception, and intentions to reduce red meat consumption.

## Methods

### Participants

In June-July 2020, we recruited a study sample using CloudResearch’s Prime Panels, a company frequently used by social and behavioral science researchers for online surveys [22].

**Fig. 1:**
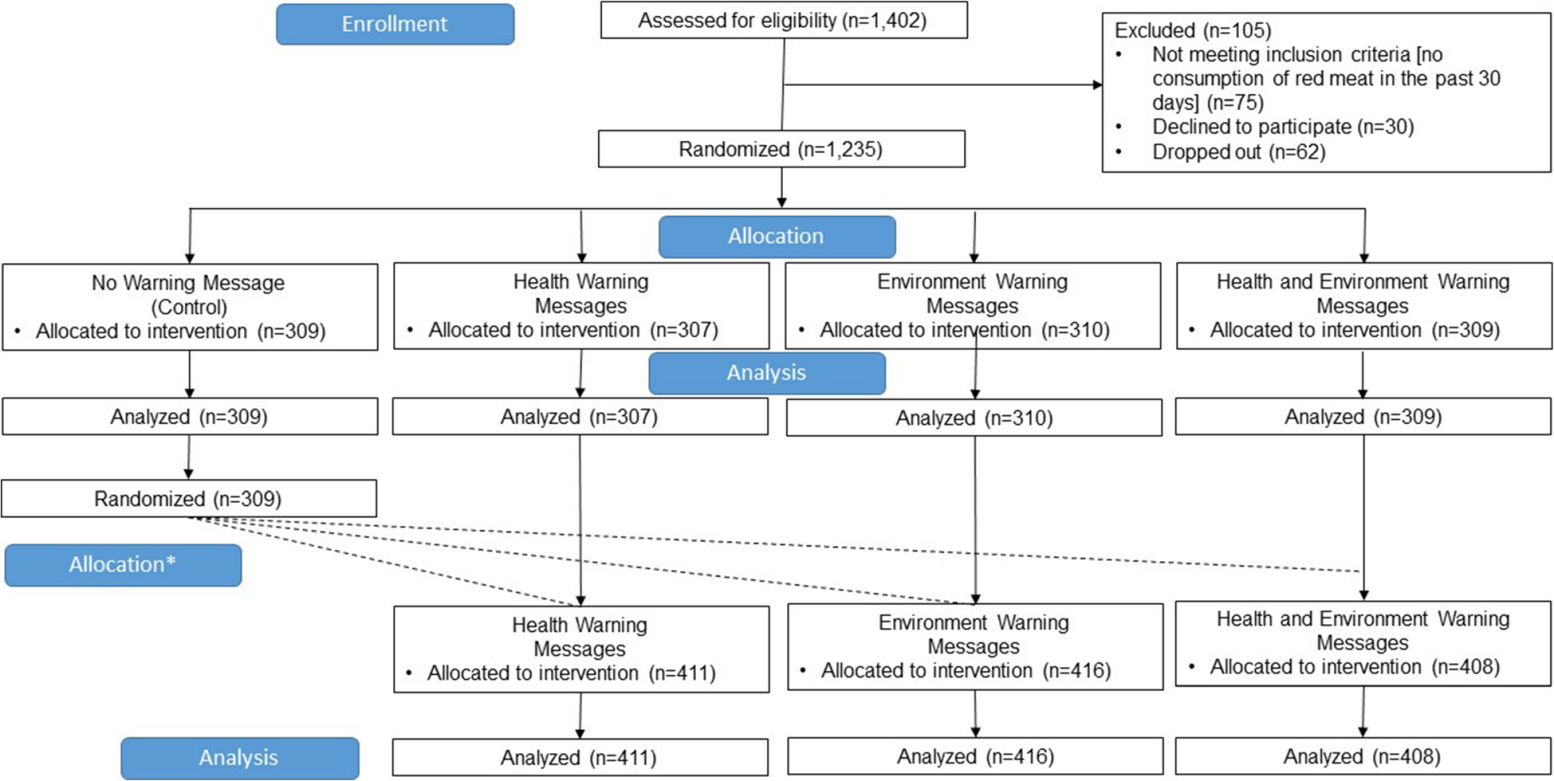
Participant flow chart. *The second experiment did not have a control condition. Participants who were randomized to the control condition for the first experiment were re-randomized to the health, environment, or combined health and environment trial arms. Participants who were randomized to health, environment, or combined health and environment for the first experiment stayed in this condition for the second experiment

CloudResearch targeted recruitment to yield a sample that was approximately representative of the US population in terms of gender, race/ethnicity, income, and age. Participants were eligible if they were 18 years or older and self-reported any red meat consumption in the past 30 days. A total of 1,235 participants completed the survey (Fig. 1). No a priori power calculation was conducted for this exploratory study.

### Warning development

The text of the warnings was developed based on a thorough review of the literature on health and environmental harms of red meat consumption. Warning messages were vetted for scientific accuracy and legal viability with an Expert Advisory Committee comprised of experts in nutrition, sustainability, food policy, agricultural policy, and law. A subset of health and environmental warning messages was then tested for perceived message effectiveness in an online pre-test experiment (paper under review) [23]. We selected warnings for this study based on the results of that pre-test experiment. First, we identified warnings with the highest perceived message effectiveness ratings. Next, because not all warnings differed significantly from one another on perceived effectiveness, we also assessed the level of evidence regarding the relationship between red meat consumption and the harm, simplicity (e.g., “carbon footprint” is simpler than “greenhouse gas emissions”), and potential political feasibility. Based on these criteria, we selected two health warnings and two environmental warnings for this study. The health warnings were: “WARNING: Eating red meat contributes to colon and rectal cancer” and “WARNING: Eating red meat increases your risk of early death.” The environmental warnings included were: “WARNING: Eating red meat increases your carbon footprint” and “WARNING: Eating red meat harms the environment.” We included the marker word, “WARNING,” based on a prior study suggesting this word may increase message effectiveness [16].

The design of the warnings was based on a study [24] that found that warnings with octagonal shape are perceived to be more effective than rectangular labels. The black octagon with white text is a commonly tested shape and color scheme in the literature on warnings [25], and this design is currently being used in countries that have government-mandated nutrient warnings on food products, including Chile, Mexico, Peru, and Uruguay.

### Product stimuli

To provide a realistic context for viewing warnings, we identified commonly consumed red meat products in the United States, using analyses of top red meat items consumed in National Health and Nutrition Examination Survey (NHANES) data [6, 26]. We also wanted to ensure there was a mix of processed and unprocessed meat products. The products included were all among the top five processed or unprocessed (respectively) red meat items in the United States: bacon, sausage, five meat pizza, cheeseburgers, ground beef, deli ham, and a beef burrito (paper under review) [27]. The beef burrito was chosen for the choice experiment because it allowed us to include similar variations (e.g., chicken and vegetarian burritos). We identified images of these products from a US online supermarket. We removed the US branding from the products and replaced it with a UK brand label (Tesco). We used an unfamiliar brand from the United Kingdom to give products a realistic appearance while minimizing the influence of brand loyalty on reactions to the warnings. Images of all products are shown in Appendix A, Additional File 1.

### Procedures

**Fig. 2:**
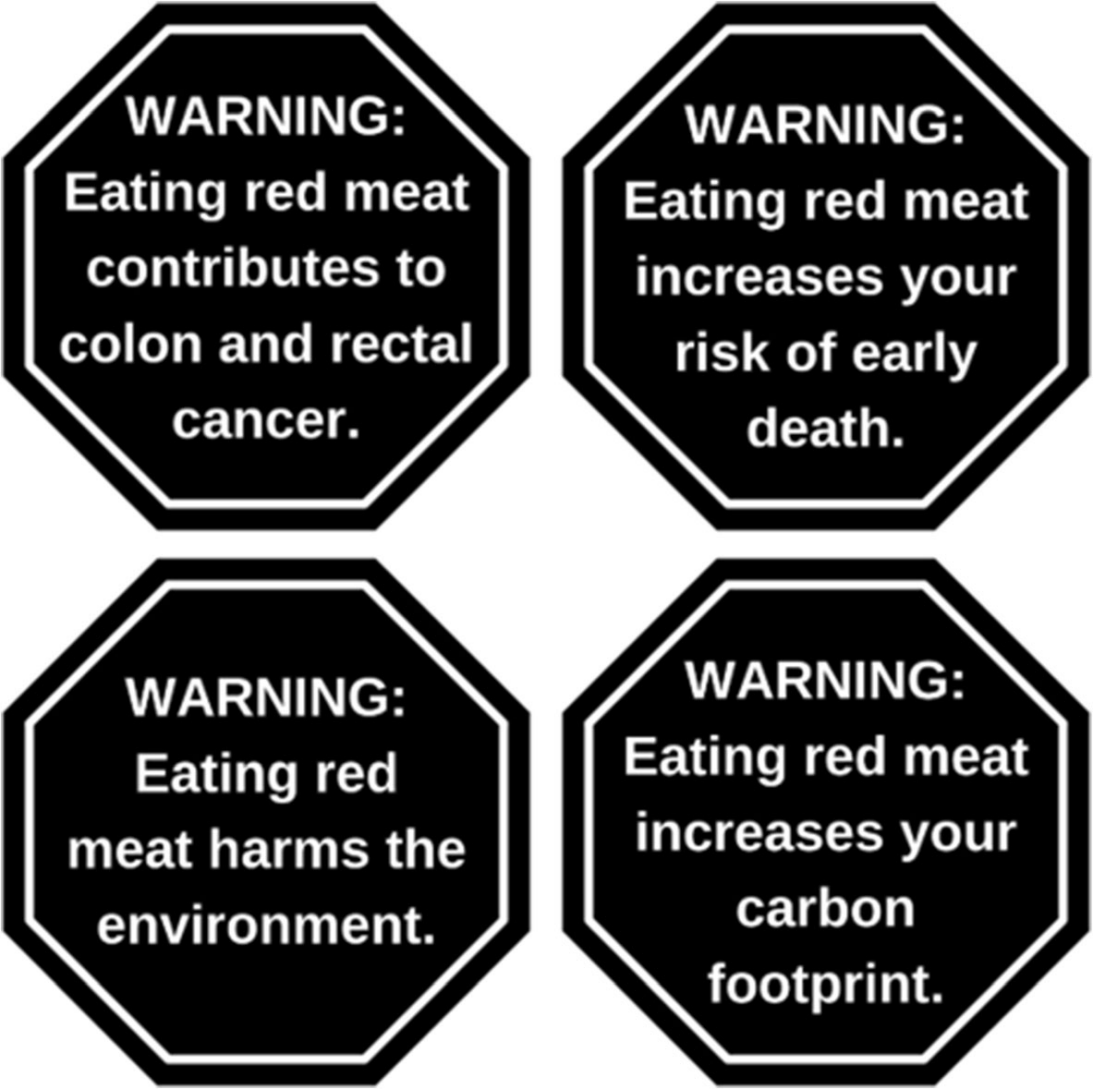
Warning labels

After providing informed consent, participants were randomly assigned following simple randomization procedures in Qualtrics to one of four conditions, in a 1:1:1:1 ratio: (1) control (no warning) [*n* = 309], (2) health warning [*n* = 307], (3) environment warning [n = 310], and (4) combined warning (health and environment) [*n* = 309] (Fig. 2). Within the health condition, participants were randomized to view one of the two warnings (colon and rectal cancer or early death). Within the environment condition, participants were randomized to view one of two warnings (carbon footprint or harms the environment). Within the combined condition, participants were randomized to one of four warning combinations, each with one health warning and one environment warning.

**Fig. 3:**
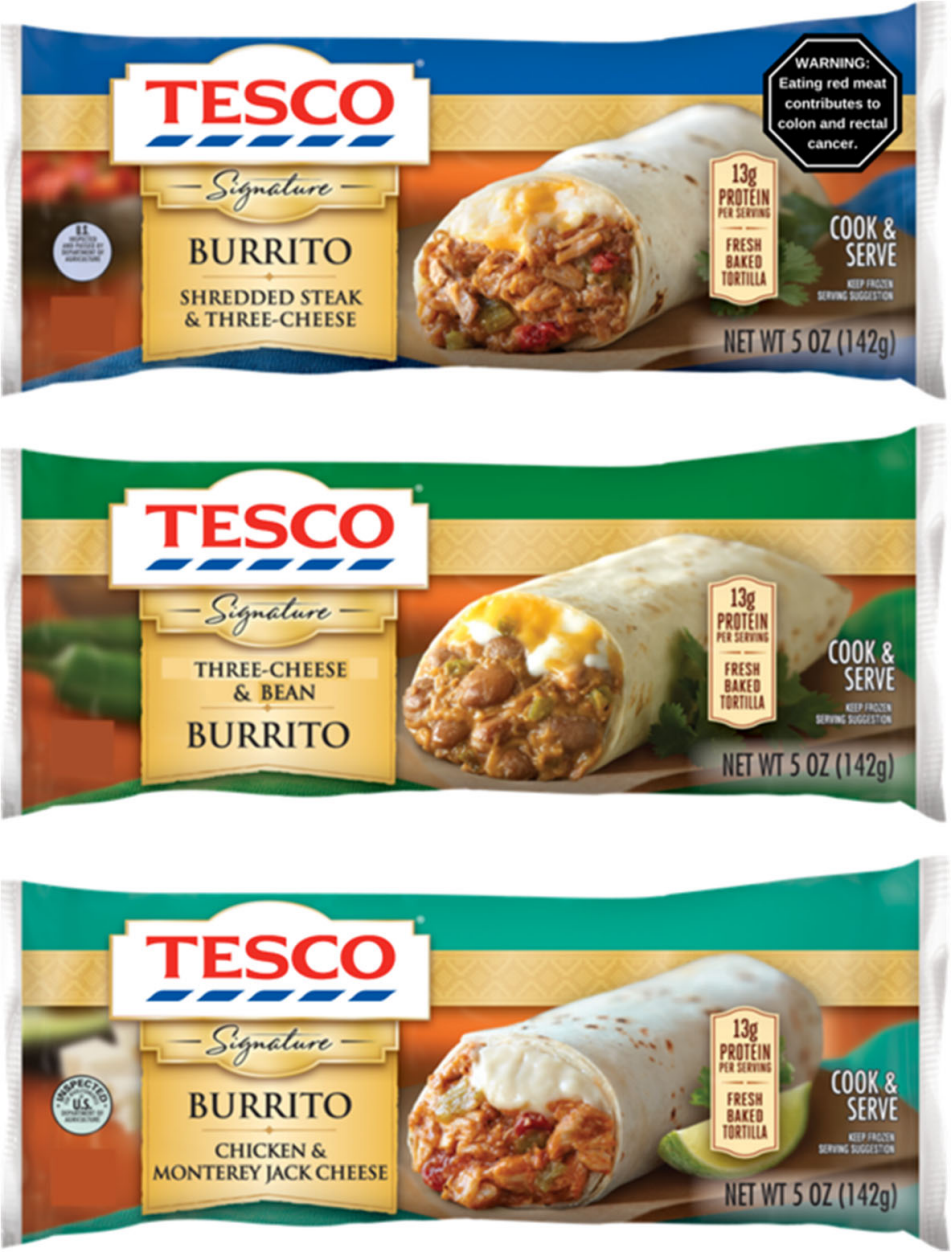
Experimental stimuli in choice task

First, participants performed a choice experiment in which they viewed three burritos (steak [red meat], chicken, or bean [Fig. 3]) in a random order. They then indicated their preferred item and which item was most harmful to health and the environment. In the warning conditions, a warning appeared on the steak burrito. Otherwise, the burritos were identical in appearance, brand, and other label information.

Second, participants viewed the warning on a series of other red meat-containing food products (Appendix A, Additional File 1). Participants in the health, environment, or combined conditions in the first experiment viewed the same warning they were assigned in the choice experiment. Participants who had been assigned to the no-warning control condition for the choice experiment were re-randomized to the health, environment, or combined warning condition. Participants viewed six red meat products (bacon, sausage, five meat pizza, cheeseburgers, ground beef, and deli ham) that displayed their assigned warning on the product packaging and then responded to questions about the warning.

After completing the experiment, participants answered questions about their demographic characteristics and belief in climate change.

## Measures

The codebook can be found in Appendix B, Additional File 1.

### Primary outcome

The primary outcome was selection of the red meat item (i.e., the steak burrito) as the item they would most like to purchase in the choice experiment. After viewing the three burritos, participants were asked, “Which of these products would you most like to buy if it was available?”

### Secondary outcomes

Secondary outcomes from the choice experiment were the selection of the steak burrito as most damaging to health and most damaging to the environment. After selecting the product they would most like to buy, participants indicated which product was the “most damaging to your health” and “the most damaging to the environment.”

We evaluated a variety of secondary outcomes about the warning labels in the second task (viewing warnings on a series of products). For those who saw both health and environment warnings, these warnings were treated as a single label rather than two separate labels, to reflect a potential policy requiring both labels. All measures used the same 5-point response scale, ranging from “Not at all” (coded as 1) to “A great deal” (coded as 5).

We assessed perceived message effectiveness using four items adapted from the University of North Carolina Perceived Message Effectiveness scale [28], which has been used effectively in previous message development studies [24, 29]. The questions were as follows: “How much does this label discourage you from wanting to eat red meat?”, “How much does this label make eating red meat seem unpleasant to you?”, “How much does this label make you concerned about the health effects of eating red meat?”, and “How much does this label make you concerned about the environmental effects of eating red meat?”

We additionally assessed warning believability (“How much do you believe these labels?”), negative emotions (three items, “How much does this label make you feel… [1] anxious, [2] scared, [3] guilty?”), and attention to the label (“How much do these labels grab your attention?”). We assessed perceptions of the health and environmental harms of red meat consumption by asking how much eating red meat every day would “increase your risk of early death,” “contribute to colon and rectal cancer,” “increase your carbon footprint,” and “harm the environment.”

Next, participants were asked if they learned anything new from the warning (“Did you learn anything new from this label?”), with response options of “Yes” or “No.” Finally, we assessed intention to reduce red meat consumption in the next 30 days (“Do you intend to reduce your red meat consumption in the next 30 days?”), on a scale of “Definitely not” (coded as 1) to “Definitely” (coded as 5).

Within each arm (health or environment), participants were asked to select which of the two health or environment warnings was most discouraging (“Which one of these labels most discourages you from wanting to consume red meat?”); participants in the combined condition saw in randomized order both health warnings and both environment warnings.

We collected data on participant demographics, including age, gender, race, ethnicity, education level, household income, and political affiliation. We also assessed participants’ red meat consumption using an item from NHANES modified to account for variations in typical intake due to the COVID-19 pandemic (“In a typical month before the COVID-19 pandemic, how often did you eat red meat?”) [30] and assessed belief in climate change (“The main cause of climate change is human activities”) [31]. Response options are available in Table 1.

**Table. 1:** United States Health and Environmental Warning Label Survey, Participant characteristics (*n* = 1,235) from June-July 2020

Characteristic	*n* = 1, 235
**Age, Mean (SD)**	44 (17)
**Gender, n (%)**	
Male	644 (53)
Female	575 (47)
**Race, n (%)**^a^	
White	939 (76)
Black or African American	123 (10)
Asian	76 (6)
Pacific Islander	6 (1)
Native American or Alaskan Native	19 (2)
Mixed Race/Multiple Races	44 (4)
Other Race Not Listed	28 (2)
**Ethnicity, n (%)**^b^	
Not Hispanic/Latinx	1,077 (87)
Hispanic/Latinx	157 (13)
**Education Level, n (%)**	
High School Diploma or Less	455 (38)
Associate or Technical Degree	249 (20)
4-year College Degree	334 (27)
Master’s, Graduate, or Higher	185 (15)
**Household Income, annual, n (%)**	
Less than $25,000	280 (23)
$25,000 to $49,999	299 (24)
$50,000 to $74,999	235 (19)
$75,000 to $99,999	162 (13)
$100,000 or more	256 (21)
**Political Affiliation, n (%)**	
Liberal	317 (26)
Moderate	524 (42)
Conservative	392 (32)
**Regular 30 Day Red Meat Consumption Frequency, n (%)**	
1 time per week or less	136 (27)
2–3 times per week	509 (41)
4 or more times per week	397 (32)
**Believe that Human Activity is Main Cause of Climate Change, n (%)**	
Strongly Disagree	92 (8)
Somewhat Disagree	91 (7)
Neither Agree nor Disagree	240 (20)
Somewhat agree	345 (28)
Strongly agree	462 (38)

^a^Race had a ‘check all that apply’ response scale. Participants that checked more than one race were summed and included in the ‘Mixed Race/Multiple Races’ category

^b^Hispanic/Latinx identity was measured separate from race and had a binary yes/no response

### Statistical analysis

All analyses were conducted in Stata/SE version 14.1 (StataCorp LLC, College Station, TX). For all analyses, *p* < 0.05 was considered statistically significant. We pre-registered analyses and hypotheses prior to data collection (https://aspredicted.org/ph7mb.pdf). All analyses were performed as specified unless otherwise noted. We reported the trial according to the CONSORT 2010 Checklist [32] (Appendix C, Additional File 1).

We used logistic regression for binary outcomes and ordinary least squares regression for continuous outcomes. Initial models included an indicator variable for assignment to the health warning, an indicator variable for assignment to the environment warning, and an interaction term between health and environment warnings (representing the combined condition). Because the interaction was not statistically significant, final models instead used indicator variables for each warning (health, environment, combined), with the control condition as the referent. For all analyses, we used the margins command to examine the differences between the outcomes between warning types.

We also examined whether two participant characteristics (belief in human-caused climate change and frequency of red meat consumption) moderated the impact of warnings on product selection by including interactions between the experimental condition (any label vs. control) and the moderator variable. We fit separate models for the two potential moderators. First, the main effects model was estimated. Then, the interaction terms were included, and a likelihood ratio test was conducted to evaluate statistical significance of the interaction. We then probed for moderation between levels using chi-square tests to examine the contrasts in the difference between any warning and no warning by level of meat consum or by level of belief in human-caused climate change.

We planned to conduct exploratory analyses examining differences in perceived message effectiveness by demographic groups, but we were unable to perform this analysis due to a technical issue in data collection whereby two demographic characteristics (age and gender) were not linked to respondent data.

## Results

A total of 1,235 participants completed the survey (Fig. 1). Participants were, on average, 44 years old (range: 18–97 years) (Table 1). About half of participants (53 %) were male and most (76 %) identified as White. Most participants (96 %) had completed high school, while 43 % had completed a college or advanced degree. Most participants (73 %) consumed red meat more than once per week.

### Choice experiment

**Fig. 4:**
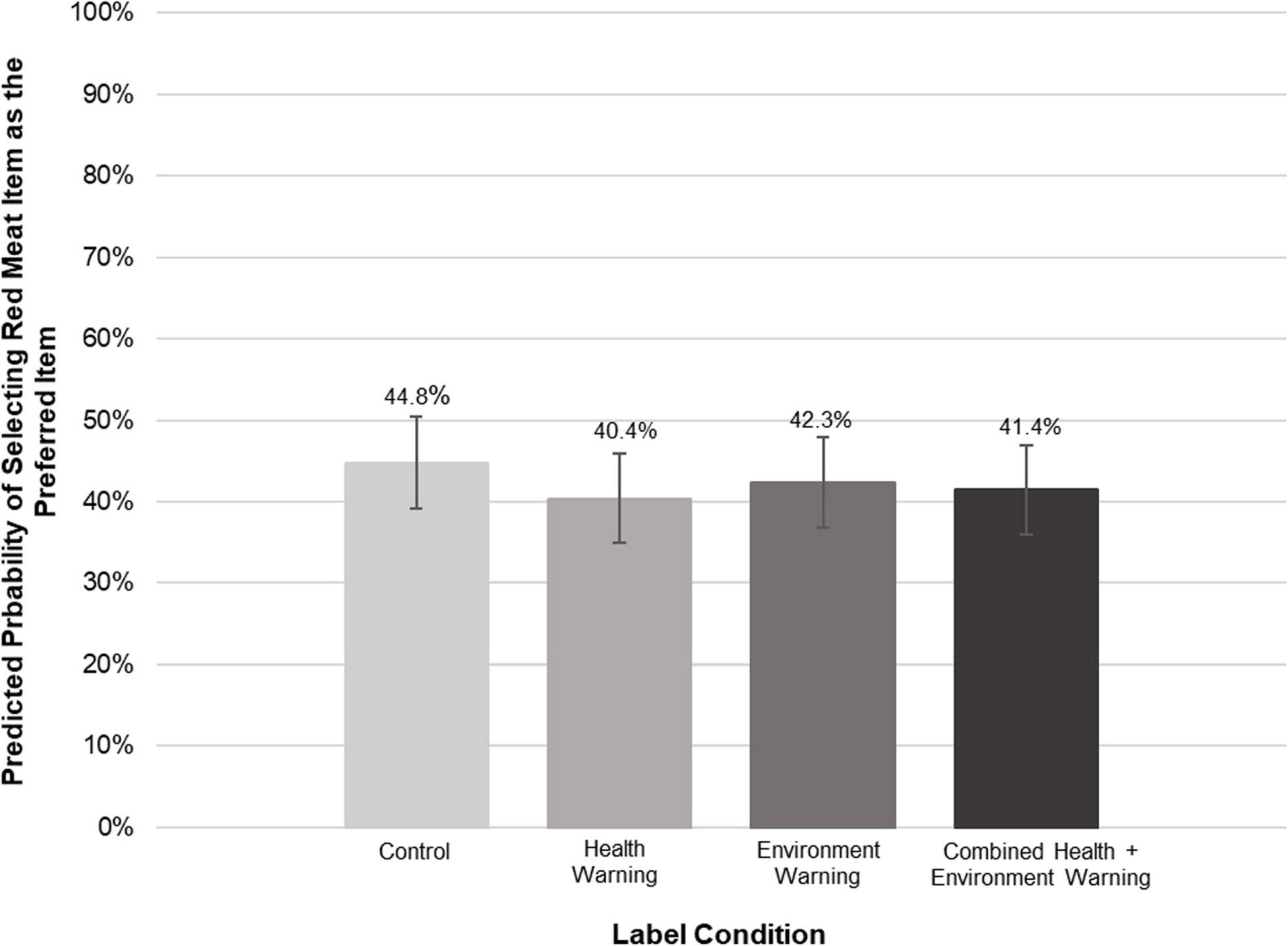
Predicted probability of participants selecting red meat item by experimental condition (*n* = 1,222). The control condition served as the referent group. The margins command was then used to examine differences between each label condition. No differences were found at *p *< 0.05. Bars represent standard errors

**Fig. 5:**
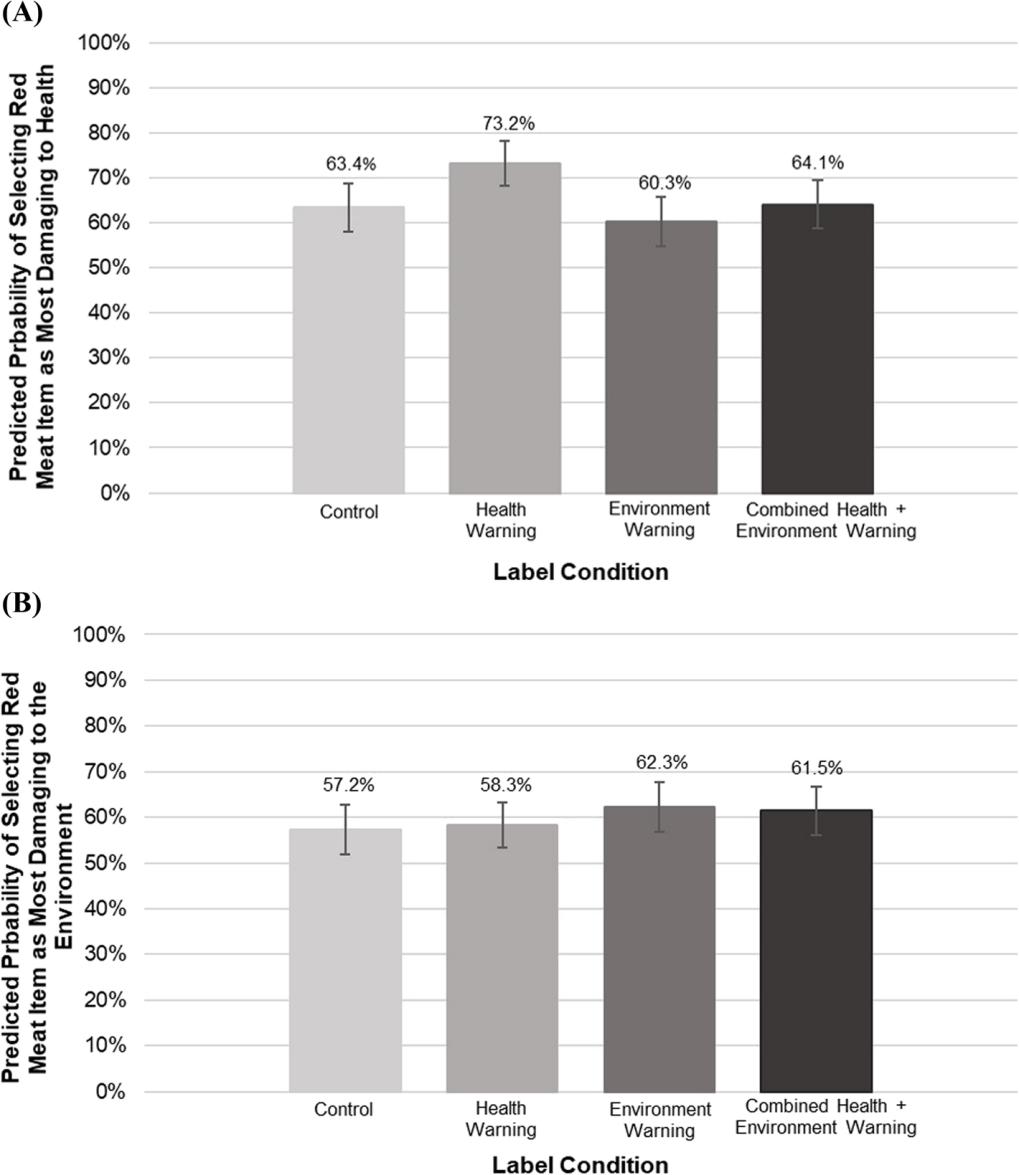
Predicted probability of participants identifying the red meat item as the item most damaging to **A**) health and **B**) the environment in an online choice experiment (*n* = 1,235). **A** The control condition served as the referent group. The margins and test commands were then used to examine differences between each label condition. Health was different than all other label conditions at *p *< 0.05. No other differences were seen between label conditions. Bars represent standard errors. **B **The control condition served as the referent group. The margins and test commands were then used to examine differences between each label condition. No differences were found at *p *< 0.05. Bars represent standard errors

In the control condition, the predicted probability of selecting the red meat item (steak burrito) as the product they would most want to buy was 44.8 %. This probability was lower in absolute value in the health warning (40.4 %), environment warning (42.3 %), or combined warning (41.4 %) conditions, but these differences were not statistically significant (Fig. 4). Participants who viewed the health warning were more likely to identify the red meat item as damaging to health compared to participants in the control arm (health label 73.2 %, no-label control 63.4 %, *p <* 0.05) (Fig. 5, Panel A). There was no significant difference in the likelihood of identifying the red meat item as most damaging to the environment, though the trend for environment and combined warnings was in the expected direction (Fig. 5, Panel B).

Although the interaction of red meat consumption and warning condition was not statistically significant (*p* = 0.08), individual contrasts showed that the impact of viewing a warning vs. no warning on the probability of selecting the beef burrito was greater by 17.6 % points for participants who reported high meat intake (four or more times per week) compared to those who reported low meat intake (one or fewer times per week) (Table S1, Additional File 1, *p* = 0.03). Belief in human-caused climate change did not modify the effect of the warnings on selection of the red meat item (Table S2, Additional File 1, *p* = 0.72), and there were no statistically significant contrasts between levels.

### Consumer reactions to warnings

Consumer reactions to warnings are presented in Table 2. Exposure to the combined and health warning performed similarly on perceived message effectiveness (mean _combined_ =2.91, mean _health_=2.84), while exposure to the environmental warning (mean _environment_=2.61,) performed worse than health (*p <* 0.001) and combined messages (*p <* 0.01). The same pattern was seen for negative emotions, where the combined and health warnings (mean _combined_ =2.49, mean _health_ =2.43) were not different from each other but elicited stronger reactions compared to the environment warning (mean _environment_ =2.24, *p <* 0.01 and *p <* 0.05 for combined and health differences, respectively). For believability, the combined warning performed better than the environment (mean _combined_ =3.10, mean _environment_ =2.93, *p* < 0.05), but was not different from the health warning (mean _health_ =3.08).

**Table 2:** Effects of warning label type consumer reactions and intentions

	Health Mean (SE)	Diff.	Environment Mean (SE)	Diff.	Combined Mean (SE)	Diff.
Perceived Message Effectiveness (*n* = 1,234)	2.84 (1.25)	A	2.61 (1.21)	B	2.91 (1.26)	A
Believability (*n* = 1,235)	3.08 (1.21)	AB	2.93 (1.24)	B	3.10 (1.19)	A
Negative emotions (*n* = 1,212)	2.43 (1.28)	A	2.24 (1.19)	B	2.49 (1.26)	A
Perceived risk						
Carbon footprint (*n* = 1,235)	3.05 (1.25)	A	3.06 (1.26)	A	3.27 (1.19)	B
Environment (*n* = 1,235)	3.07 (1.24)	AB	2.94 (1.27)	A	3.22 (1.21)	B
Early death (*n* = 1,234)	3.31 (1.22)	A	3.04 (1.14)	B	3.30 (1.21)	A
Colon and rectal cancer (*n* = 1,235)	3.33 (1.21)	A	3.03 (1.17)	B	3.36 (1.14)	A
Grabs attention (*n* = 1,235)	3.54 (1.21)	A	3.32 (1.17)	B	3.32 (1.21)	B
Intentions to reduce (*n* = 1,234)	2.85 (1.39)	A	2.79 (1.36)	A	2.91 (1.32)	A
	%	Diff.	%	Diff.	%	Diff.
Learned something new (*n* = 1,235)	52	AB	46	A	58	B

*Note. L*earning something new was a binary Yes/No outcome. Percents presented are % that responded “yes” to learning something new. All other outcomes were measured on a 5 point scale. Intentions to reduce was measured as (1=Definitely not...5 Definitely). The other outcomes were measured on a scale of 1=Not at all...5=A great deal)

*SE* standard error, *Diff* Within each row, conditions sharing a letter are not significantly different from each other at *p*<0.05

Those in the combined warning condition were more likely to report they learned something new from their warning compared to those in the environment warning condition (predicted probability 58 % versus 46 %, respectively, *p <* 0.01), but were not statistically different than the health warning (predicted probability: 52 %).

The combined and health warnings had similar impacts on perceived risk of colon and rectal cancer (mean _combined_ =3.36, mean _health_=3.33) and early death (mean _combined_ =3.30, mean _health_=3.31), and were not different from each other. Both warnings outperformed the environment warning on these outcomes (means 3.04 and 3.03 for early death and colon and rectal cancer, respectively, both *p <* 0.01). For perceived harms of carbon footprint, the combined warning (mean _combined_ =3.27) performed better than both health and environment warnings (mean _health_ =3.05, mean _environment =_3.06, *p <* 0.05), while health and environment warnings were not different from one another. For perceived environmental damage, the combined warning (mean _combined_ =3.22) performed better than the environment warning (mean _environment_ =2.94, *p <* 0.01). The health warning (mean _health_ =3.07) was not different from either other condition.

The health warning outperformed the combined warning on only one outcome, attention. Specifically, the health warning (mean _health_ =3.54) elicited more attention than the combined (mean _combined_ =3.32) and environment (mean _environment_ =3.32) warnings (*p <* 0.05), while the environment and combined warnings were not different from each other. There were no differences between warnings on intentions to reduce red meat consumption.

Within health warnings, a higher proportion of participants selected the warning about colon and rectal cancer as more discouraging than the early death warning (Figure S1). Within environmental warnings, a higher proportion of participants selected the harms the environment warning as more discouraging than the carbon footprint warning (Figure S2).

## Discussion

In this online experiment of US adults, we found that health, environment, and combined warnings about red meat did not reduce participants’ selection of red meat as their preferred item of choice. The health warning, but not the other warnings, increased participants’ likelihood of identifying the red meat item as the most damaging to health. Health warnings and combined health and environment warnings outperformed environment-only warnings on several consumer reactions, including perceived message effectiveness, believability, negative emotions, and perceived health harms.

It is unclear why warnings did not reduce consumers’ selection of the red meat item (steak burrito). Literature from sugar-sweetened beverages shows that both simple nutrient warnings (e.g., “high in sugar”) and health warnings reduce consumers’ selection of high-sugar food and drink [16, 24, 25]. One possibility is that underlying preferences for red meat are stronger than they are for sugar-sweetened beverages. For example, in the 2015–2016 NHANES survey, 47.8 % of US adults were sugar-sweetened beverage consumers [33], while in 2013–2016 NHANES, 73.6 % of US adults were red meat consumers [34].

In addition, it is important to consider the role of substitutes: making a choice to decline a particular product necessitates a decision to purchase an alternate product and may depend on how much consumers prefer or value the alternate option. It is possible that the substitutions for the steak burrito were less likely to be accepted by consumers than are substitutions for sugar-sweetened beverages. Substitutions for sugar-sweetened beverages are similar: substitutions are often other sweet drinks that are sweetened with something other than sugar (for example, consumers might choose between a fruit drink sweetened with sugar versus a fruit drink sweetened with non-caloric sweetener). In contrast, substitutions for the steak burrito in the current experiment were more dissimilar in taste profiles (e.g., a steak burrito versus one that contains chicken or beans). Plant-based biomimicry products (e.g., Beyond Meat) may be a more acceptable substitution than an alternate animal protein or vegetarian option for consumers with high red meat preferences. In addition, the effect of warnings on red meat selection could vary across product categories (e.g., the effect of a warning could be stronger for products containing more red meat, such as a burger patty, than for products that contain less red meat as part of a mixed dish, such as a burrito). More research is needed to understand whether warnings shift consumers’ selections across product categories, and how this varies by what alternate options are available.

While the warnings did not reduce selection of the red meat item in the sample overall, we found a pattern of results that suggested that warnings’ impact on product selection was stronger among those who consumed more meat. In general, there is a dearth of evidence on whether those who consume more of a product are more or less responsive to warning labels; more research is specifically needed to understand whether more frequent meat-consumers would actually change their behavior in response to warnings. If this was the case, these results would be in line with other point-of-purchase policies, primarily sugar-sweetened beverages and nonessential food taxes, which have found that those who consume more taxed products reduce their purchases more [35, 36]. If policies such as warnings have a greater impact among more frequent meat-consumers, this could yield large health and environmental benefits, given these individuals have the largest room for improvement in their dietary behaviors.

When considering consumer reactions to specific types of warnings, including perceived message effectiveness, perceived risk, and learning something new from the warning, the combined warning generally performed the best, followed by the health warning, then the environment warning, though not all differences between warnings were statistically significant. Previous nationally representative surveys have found that among meat-reducers and vegetarians, health is a more important factor than environment in reducing meat consumption [37, 38]. The findings from this study show a similar pattern among red meat consumers in which health harms may be more motivating than environmental harms. It is also possible that awareness of the environmental harms of red meat is not as widespread as awareness of the health harms of red meat. As public awareness of these harms increases, it is possible that warning messages will elicit stronger responses [39]. Combined warning messages may be a win-win in that they elicit stronger responses from the health warning while simultaneously raising awareness of red meat’s environmental harms.

On the other hand, the impact of the warnings did not vary by belief in climate change. This finding was unexpected, as we predicted that the environment warning in particular would have a larger effect among those with pre-existing beliefs in climate change, as previous surveys have found those with stronger climate change beliefs are more willing to reduce their meat consumption [38]. However, prior surveys have asked about willingness to reduce meat consumption generally, rather than assessing preferences in a choice experiment. It may be that people who believe in climate change are generally open to the idea of changing their meat consumption but are not yet ready to implement those behaviors. Future research integrating the Stages of Change model would be useful to understand the most effective strategies for moving meat consumers from contemplation to behavioral change [40].

The finding that environment warnings did not reduce selection of red meat was somewhat surprising given previous literature showing that other environment-related labels in real-world food environments reduced purchases of less sustainable products [41] and decreased purchases of meat [42]. These differences could be due to differences in label type (e.g., warnings vs. a carbon-label footprint) or in population, as most previous studies have been conducted in Europe, where populations may be more receptive to environmental messaging. Interestingly, the Vanclay et al. study, which tested carbon footprint labels that were color-coded to signal low (green), medium (yellow), and high (black) carbon emissions found a bigger reduction in black-to-green purchases when green-labeled products were the cheapest [41], suggesting that combing labeling with pricing strategies may increase labels’ impact. Future research on reducing meat consumption should consider combining pricing policies (taxes or subsidies) to understand how these policies might interact in the US context.

It is also worth noting that, within environment warnings, the majority of participants selected the warning about harming the environment as more discouraging than the carbon footprint warning. If the carbon footprint warning was indeed less effective, this could have dampened the overall effect of environment-related warnings on product selection. Similarly, of those who viewed the health warning, the majority selected the warning about colon and rectal cancer as more discouraging than the warning about death. This suggests that the specific warning topic could influence warnings’ impact on behavior, and merits more research into understanding which topics will resonate most with consumers.

The most important limitation of this study was that it was a one-time online experiment with a limited number of products. Our findings may not translate directly to real-world settings where individuals are exposed to warning labels on many different products, can pick products up to examine them, and see warnings repeatedly over time. Although we assessed whether the labels grabbed participants’ attention, we did not explicitly assess whether participants noticed the warning or the length of time spent viewing each image. These data should be collected in future studies. While this study is best suited to provide future researchers with data on how to design effective warnings, it does provide some insights into what might be expected in the real-world, especially considering that online grocery purchases have more than doubled in the past year as a result of the COVID-19 pandemic [43].

An additional limitation is that the lack of differences between study conditions may have been due to insufficient power to detect small reductions in selection likelihood, rather than a true lack of effectiveness.

Strengths of the study include the large sample that approximated the US population in terms of gender, race/ethnicity, income, and age. Another major strength was the experimental design; by randomizing participants, we minimized the effect of potential confounders. We also assessed a range of outcomes using measures with strong psychometric properties to capture different aspects of consumer reactions to warnings.

## Conclusions

A growing body of work is focused on policies and interventions to reduce red meat intake as a strategy to reduce chronic disease and mitigate climate change. This study did not find a significant effect of health or environment warnings on consumer preference for a red meat item (steak burrito) in a choice experiment, though effects were in the expected direction. When comparing warning types, the combined health and environment warnings and health-only warnings performed better than the environment-only warning across most consumer reactions, including perceived message effectiveness. Future research should evaluate the impact of warnings in realistic settings and on actual consumer behaviors, as well as evaluate a broader range of potential health and environmental harms to highlight in communication campaigns.

## Supplementary Information

**Additional file 1: Appendix A. **Sample red meat products from second experiment. **Table S1.** Selection of Steak Burrito as the Preferred Item Predicted by Viewing a Warning and Red Meat Consumption. **Table S2.** Selection of Steak Burrito as the Preferred Item Predicted by Viewing a Warning and Belief in Climate Change. **Figure S1. **Health Warning Identified as Most Discouraging Red Meat Consumption by Participants Exposed to Health Warnings (*n *= 803). **Figure S2. **Environmental Warning Identified as Most Discouraging Red Meat Consumption by Participants Exposed to Environment Warnings (*n *= 811). **Appendix B.** Codebook. **Appendix C.** CONSORT 2010 Checklist.

## Data Availability

Data requests will be handled on a case-by-case basis due to IRB policies. Please contact the corresponding author to request access to the dataset.
