# Supplementary material for: Testing front-of-package warnings to discourage red meat consumption: a randomized experiment with US meat consumers
Source: Int J Behav Nutr Phys Act. 2021 Sep 8;18:114. doi: 10.1186/s12966-021-01178-9 (PMC8423585; doi:10.1186/s12966-021-01178-9)
Supplement: Supplementary file 1 — Additional file 1: Appendix A. Sample red meat products from second experiment. Table S1. Selection of Steak Burrito as the Preferred Item Predicted by Viewing a Warning and Red Meat Consumption. Table S2. Selection of Steak Burrito as the Preferred Item Predicted by Viewing a Warning and Belief in Climate Change. Figure S1. Health Warning Identified as Most Discouraging Red Meat Consumption by Participants Exposed to Health Warnings (n = 803). Figure S2. Environmental Warning Identified as Most Discouraging Red Meat Consumption by Participants Exposed to Environment Warnings (n = 811). Appendix B. Codebook. Appendix C. CONSORT 2010 Checklist. [file 12966_2021_1178_MOESM1_ESM.docx]

**Appendix A.** Sample red meat products from second experiment.


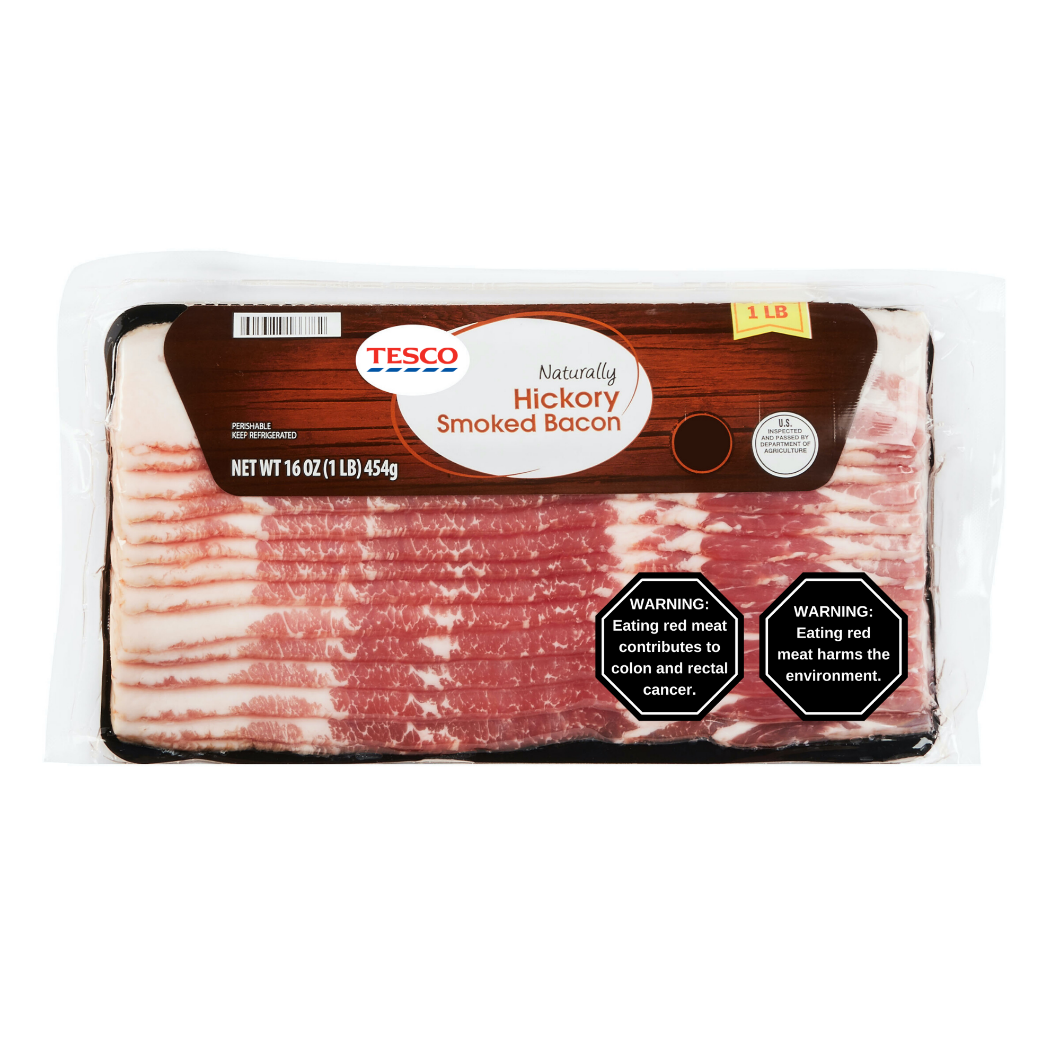

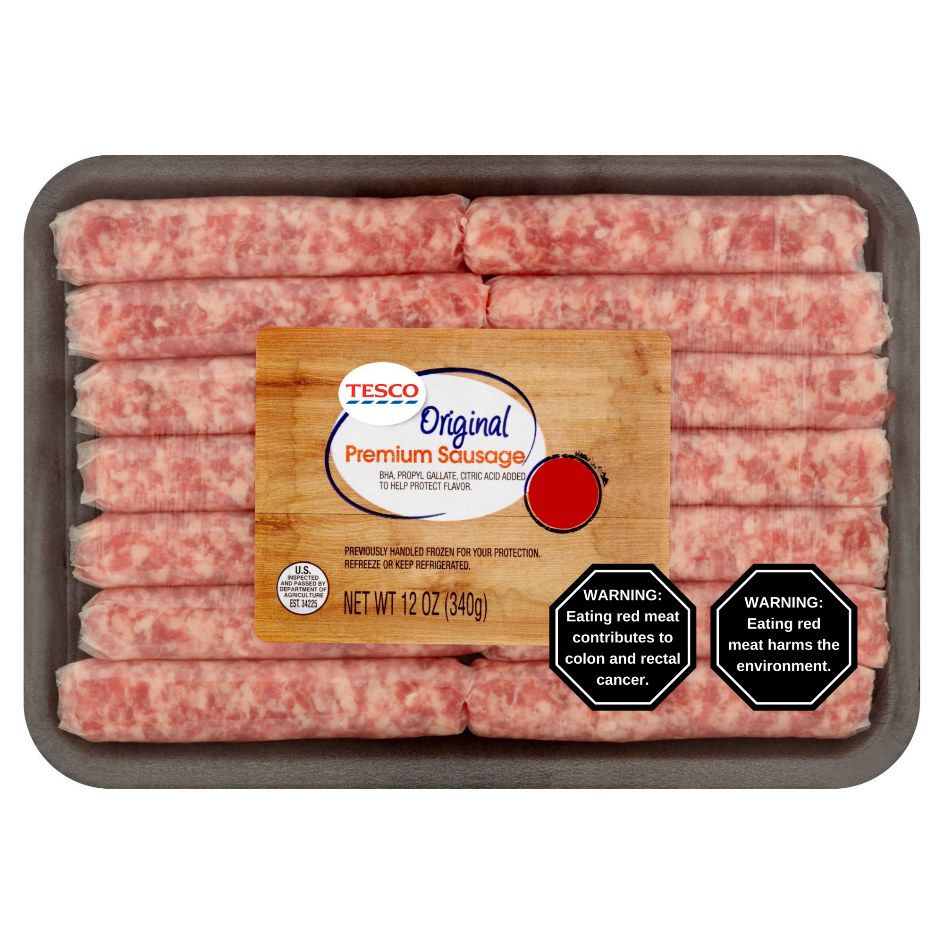

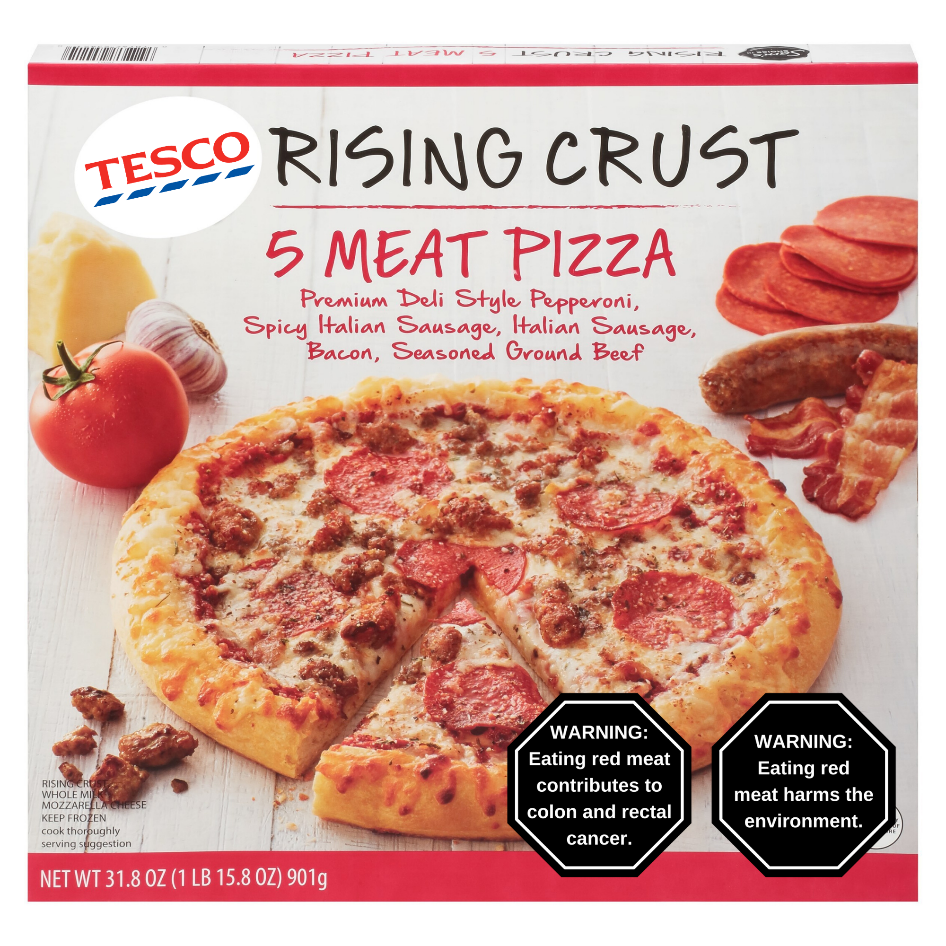

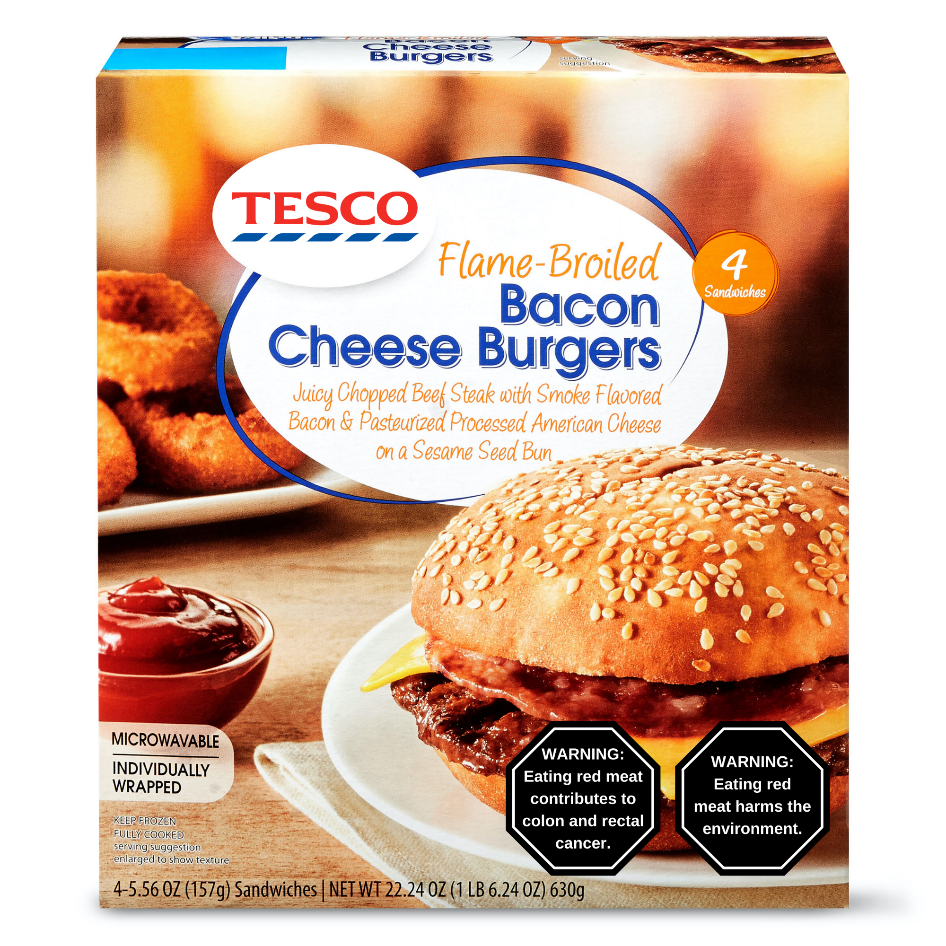

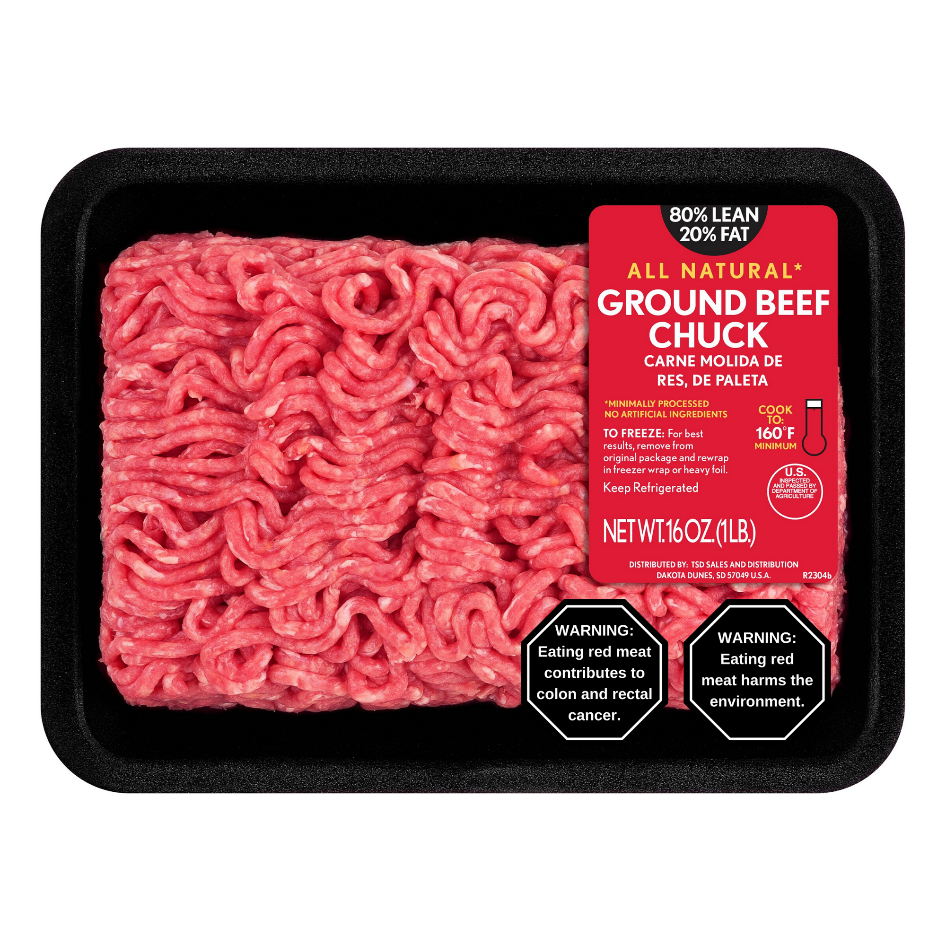

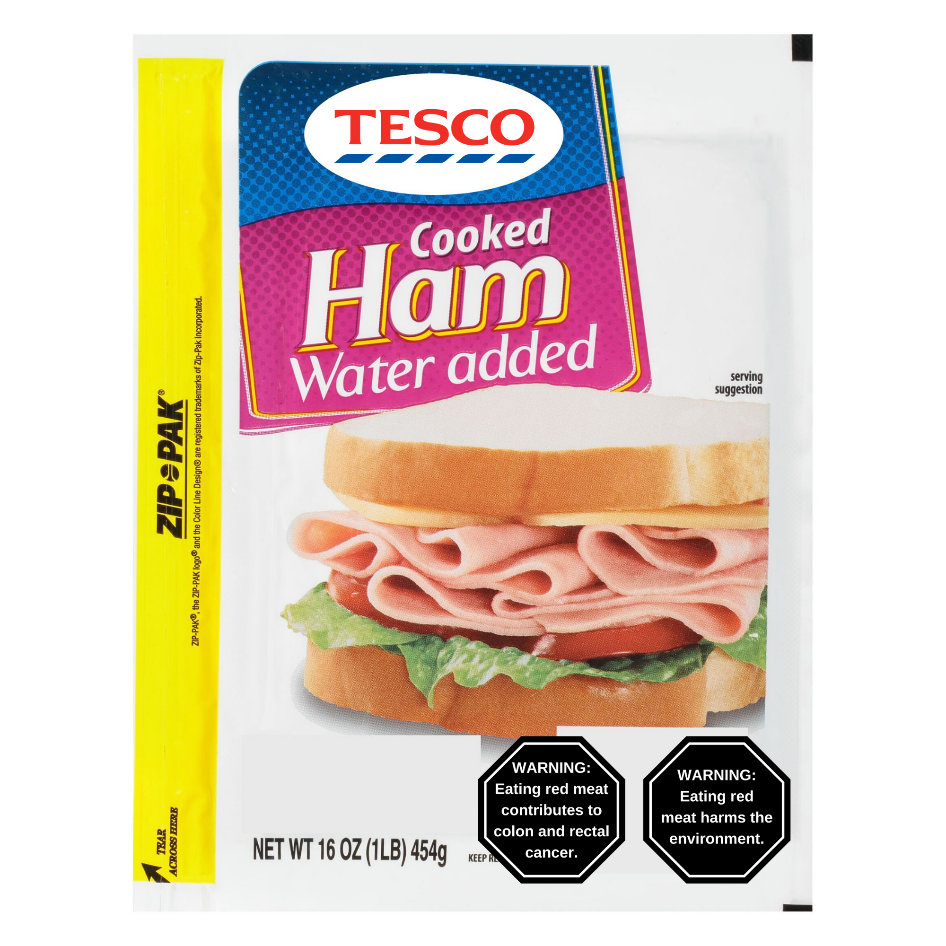


**Table S1.** Selection of Beef Burrito as the Preferred Item Predicted by Viewing a Warning Label and Red Meat Consumption

| Red Meat Consumption | Contrast (pp) | 95% CI | | p-value |
| --- | --- | --- | --- | --- |
| 1 time per week or less | ref | -- | -- | -- |
| 2-3 times per week | -3.8 | -18.8 | 11.2 | 0.62 |
| 4 or more times per week | -17.6 | -33.6 | -1.6 | 0.03 |

^a^Results are for the contrast of the effect of any warning vs. no warning on the probability of selecting the beef burrito between red meat consumption levels (pp = percentage points).
Likelihood ratio test for interaction p=0.085
Note: all three warning conditions were collapsed due to there being no significant differences between conditions.

**Table S2.** Selection of Beef Burrito as the Preferred Item Predicted by Viewing a Warning Label and Belief in Climate Change

| Belief in Climate Change | Contrast (pp) | 95% CI | | p-value |
| --- | --- | --- | --- | --- |
| Somewhat or strongly disagree | ref | -- | -- | -- |
| Neither agree not disagree | -3.6 | -26.4 | 19.2 | 0.76 |
| Somewhat agree | 0.5 | -20.1 | 22.1 | 0.96 |
| Strongly agree | -8.1 | -28.6 | 12.4 | 0.44 |

^a^Results are for the contrast of the effect of any warning vs. no warning on the probability of selecting the beef burrito between levels of belief in climate change (pp = percentage points).
Likelihood ratio test for interaction p=0.72
Note: all three warning conditions were collapsed due to there being no significant differences between conditions.

**Figure S1.** Health Label Identified as Most Discouraging Red Meat Consumption by Participants Exposed to Health Labels (n=803).

**Figure S2.** Environmental Label Identified as Most Discouraging Red Meat Consumption by Participants Exposed to Environment Labels (n=811)

| **Appendix B.** Codebook. | | |
| --- | --- | --- |
| **# con**  Variable description | **Item**  Question text  [note that all items will request (but not force, except where noted) a response. Instructions to programmers are in brackets] | **Response**  **Response scale and note to programmers**  **Note for missing:** All missing values are coded as ‘99’. |
| Red meat screener  Modified NHANES  screener | **In a typical month before the COVID-19 pandemic, how often did you eat red meat?**      Red meat includes beef, lamb, pork, sausage, and ham. It also includes processed red meats such as bacon, hot dogs, and lunch meats. It **does not include** chicken, turkey, or seafood products.  [show image of red meats] | **Note to programmers: You do not need to program this item. It is what we will use as the Prime Panel exclusion question.**  0=Never  1=Less than 1 time per week  2=1 time per week  3=2-3 times per week  4=4-6 times per week  5=1 time per day  6=2 times per day  7=3 or more times per day |
|  | **I: Initial prompts and questions** |  |
| consent | Consent form will be shown (attached with IRB as separate document). At the bottom of the consent form, it states “**By continuing on to the survey below, you acknowledge that you have read and understand the information on this page and agree to be in this research study. Thank you!”** |  |
| I30 | **Please read each question carefully. You will not be able to change your answers after you advance to the next page.** |  |
|  | 1. **Choice Experiment** |  |
|  | [Randomize people to one of 4 arms.]  Arm 1: Control  Arm 2: Health Label  Randomize in equal numbers to:  Health 1: WARNING: This product contributes to colon and rectal cancer.  Or Health 2: WARNING: This product increases your risk of early death.  Arm 3: Environment label.  Randomize in equal numbers to:  Environment Label 1 text: WARNING: This product increases your carbon footprint.  Environment Label 2: WARNING: This product harms the environment.  Arm 4: Health+ environment  Health 1 + Env 1  Health 1 + Env 2  Health 2 + Env 2  Health 2 + Env 1 |  |
| A10  prompt | [one-time prompt]:  **You will now see some food products on the following screens and answer some questions about them.**  [See Appendix for product images (burritos)] | **Note for programmers:** Show the prompt. Then, show the products in random order, each product on a new page. Then, proceed to A20. Burrito will include the warning message for their respective condition. |
| A20  Choice task-purchase preference  preference | Which of these products would you most like to buy if it were available?  [show images of all 3 burritos side-by-side]. | 1=Beef  2=Chicken  3=Bean  [maintain initial randomization of images] |
| A30  Choice task-health  damage_health | Which of these products is the most damaging to your health? | 1=Beef  2=Chicken  3=Bean  [maintain initial randomization of images] |
| A40  Choice task-environment  damage_enviro | Which of these products is the most damaging to the environment? | 1=Beef  2=Chicken  3=Bean  [maintain initial randomization of images] |
|  | 1. **Main Experiment: Meat Messages** |  |
|  | [Condition from choice experiment should be carried over, except for those in the control group. Control group subjects should be re-randomized. So, the three conditions are:  Arm 1: Health  Aims 2: Environment  Arm 3: Health + environment] |  |
| B10  Prompt  Grummon et al. (2019) | [prompt, first message]:  **You will now look at a label. We will show you the label on different products. Then, you will see the label by itself and answer questions about that label.** [Insert page break] |  |
|  | 1. **Main message experiment** |  |
| C10.  Images | [Participants will see the same label they saw in the choice experiment. They will see the label on 6 products and answer questions about that label.  Health Label 1: WARNING: Eating red meat contributes to colon and rectal cancer.  Health Label 2: WARNING: Eating red meat increases your risk of early death.  Environment Label 1: WARNING: Eating red meat increases your carbon footprint.  Environment Label 2: WARNING: Eating red meat harms the environment.  [Show each image on a separate page]  **[See Teams gallery for product images – bacon, sausage, pizza, cheeseburger, ground beef, lunch meat]** | **Note to programmers:**  Randomize the order of the images. Show all the images and then show the questions. |
| C20  attention | [Show all images side by side]  [Show enlarged label]  This label was on the products you just saw. How much does this label grab your attention?  (Combined condition: These labels were on the products you just saw. How much do these labels grab your attention?)  [page break] | **Note to programmers: For the combined condition, show both labels the participant viewed and change ‘this label’ to ‘these labels’.**  1 = Not at all  2 = Very little  3 = Somewhat  4 = Quite a bit  5 = A great deal |
| C30  PME: Discouragement  Baig et al. (2019)  Grummon et al. (2019)  pme_discourage | How much does this label discourage you from wanting to eat red meat?  (Combined condition: How much do these labels discourage you from wanting to eat red meat?) | **Note to programmers: Randomize order of the 4 PME items. All 4 questions should appear on the same page. Show labels at the top of the page.**    1 = Not at all  2 = Very little  3 = Somewhat  4 = Quite a bit  5 = A great deal |
| C40  PME: Unpleasantness  Baig et al. (2019)  Grummon et al. (2019)  pme_unpleasant | How much does this label make eating red meat seem unpleasant to you?  (Combined condition: How much do these labels make eating red meat seem unpleasant to you?) | 1 = Not at all  2 = Very little  3 = Somewhat  4 = Quite a bit  5 = A great deal |
| C50  PME: Concern  Baig et al. (2019)  Grummon et al. (2019)  pme_health | How much does this label make you concerned about the health effects of eating red meat?  (Combined condition: How much do these labels make you concerned about the health effects of eating red meat?) | 1 = Not at all  2 = Very little  3 = Somewhat  4 = Quite a bit  5 = A great deal |
| C60  PME: Concern  Baig et al. (2019)  Grummon et al. (2019)  pme_enviro | How much does this label make you concerned about the environmental effects of eating red meat?  (Combined condition: How much does these labels make you concerned about the environmental effects of eating red meat?)  [page break] | 1 = Not at all  2 = Very little  3 = Somewhat  4 = Quite a bit  5 = A great deal |
| C90  Negative emotions-anxious  neg_anxious | How much does the label make you feel anxious?  (Combined condition: How much do these labels make you feel anxious?) | **Note to programmers: Randomize order of the 3 negative affect items. All 3 questions should appear on the same page. Display label at the top of the page.**  1 = Not at all  2 = Very little  3 = Somewhat  4 = Quite a bit  5 = A great deal |
| C100  Negative emotions-scared  neg_scared | How much does this label make you feel scared?  (Combined condition: How much do these labels make you feel scared?) | 1 = Not at all  2 = Very little  3 = Somewhat  4 = Quite a bit  5 = A great deal |
| C110  Negative emotions-guilt  neg_guilt | How much does this label make you feel guilty?  (Combined condition: How much do these labels make you feel guilty?) | 1 = Not at all  2 = Very little  3 = Somewhat  4 = Quite a bit  5 = A great deal |
| C120  Believability  believe | How much do you believe this label?  (Combined condition: How much do you believe these labels?) | 1 = Not at all  2 = Very little  3 = Somewhat  4 = Quite a bit  5 = A great deal |
| C130  Learning  learn | Did you learn anything new from this label?  (Combined condition: Did you learn anything new from these labels?) | 1=Yes  0=No |
| C140  Most discouraging label  most_discourage | [page break]  Which one of these labels most discourages you from wanting to consume red meat? | **Note to programmers:**  Show this question after the participant has seen both warning labels and answered all the questions through C130. For those in the combined condition, show this questions twice; one for health and one for environment. Randomize the order.  1=Message 1  2=Message 2 |
|  | **P: Perceived Risks of Climate Change/Health** |  |
| Prompt | **The next questions are about eating red meat.** | **Randomize the order of the next 4 questions.** |
| P10  Perceived risk-death  risk_death | How much would eating red meat every day increase your risk of early death? | 1 = Not at all  2 = Very little  3 = Somewhat  4 = Quite a bit  5 = A great deal |
| P20  Perceived risk-cancer  risk_cancer | How much would eating red meat every day contribute to colon and rectal cancer? | 1 = Not at all  2 = Very little  3 = Somewhat  4 = Quite a bit  5 = A great deal |
| P30  Perceived risk – carbon footprint  risk_carbon | How much would eating red meat every day increase your carbon footprint? | 1 = Not at all  2 = Very little  3 = Somewhat  4 = Quite a bit  5 = A great deal |
| P40  Perceived risk-environment  risk_enviro | How much would eating red meat every day harm the environment? | 1 = Not at all  2 = Very little  3 = Somewhat  4 = Quite a bit  5 = A great deal |
| P50  Intentions to limit meat consumption  Adapted from Malek et al (2019)  reduce_meat | Do you intend to reduce your red meat consumption in the next 30 days? | 1=Definitely not  2=Probably not  3=Possibly  4=Probably  5=Definitely |
|  | **GMO** |  |
| G0 Prompt | **Please tell us how much you agree or disagree with the following statements:** |  |
| G01  gmo_willing_cows | I am willing to consume hamburger from cows. | 1=Strongly disagree  2=Disagree  3=Neither disagree or agree  4= Agree  5= Strongly agree |
| G02  gmo_disgust_cows | I am disgusted by consuming hamburger from cows. | 1=Strongly disagree  2=Disagree  3=Neither disagree or agree  4= Agree  5= Strongly agree |
| G03  gmo_unnatural_cows | I think hamburger from cows is unnatural. | 1=Strongly disagree  2=Disagree  3=Neither disagree or agree  4= Agree  5= Strongly agree |
| G10  gmo_willing_gm | I am willing to consume hamburger from genetically modified (GM) cows. | 1=Strongly disagree  2=Disagree  3=Neither disagree or agree  4= Agree  5= Strongly agree |
| G20  gmo_disgust_gm | I am disgusted by eating hamburger from genetically modified (GM) cows. | 1=Strongly disagree  2=Disagree  3=Neither disagree or agree  4= Agree  5= Strongly agree |
| G30  gmo_unnatural_gm | I think hamburger from genetically modified (GM) cows is unnatural. | 1=Strongly disagree  2=Disagree  3=Neither disagree or agree  4= Agree  5= Strongly agree |
| G40  Prompt | Biotechnologists are working on genetically modified (GM) dairy cows that are hornless by inserting a gene from a different species of cattle. This prevents farmers from having to dehorn the cows at a young age, which can be painful to the cows. It also makes the cows safer for humans to handle on the farm.  Please tell us how much you agree or disagree with the following statements: |  |
| G50  gmo_willing_hornless | I am willing to consume hamburger from hornless genetically modified (GM) cows. | 1=Strongly disagree  2=Disagree  3=Neither disagree or agree  4= Agree  5= Strongly agree |
| G60  gmo_disgust_hornless | I am disgusted by eating hamburger from hornless genetically modified (GM) cows. | 1=Strongly disagree  2=Disagree  3=Neither disagree or agree  4= Agree  5= Strongly agree |
| G70  gmo_unnatural_hornless | I think hamburger from hornless genetically modified (GM) cows is unnatural. | 1=Strongly disagree  2=Disagree  3=Neither disagree or agree  4= Agree  5= Strongly agree |
| G80 Prompt  (Wilks, M., & Phillips, C. J. 2017). | Biotechnologists are using genetically modified (GM) yeast in the laboratory to make a plant protein that tastes like meat. The protein is then extracted from the yeast and mixed with oils and starch to make hamburger. No animal flesh is used for this lab-based hamburger and it tastes the same as hamburger from cows.  Please tell us how much you agree or disagree with the following statement: |  |
| G90  gmo_willing_yeast | I am willing to consume hamburger made with plant proteins from genetically modified (GM) yeast. | 1=Strongly disagree  2=Disagree  3=Neither disagree or agree  4= Agree  5= Strongly agree |
| G100  gmo_disgust_yeast | I am disgusted by eating hamburger made with plant proteins from genetically modified (GM) yeast. | 1=Strongly disagree  2=Disagree  3=Neither disagree or agree  4= Agree  5= Strongly agree |
| G110  gmo_unnatural_yeast | I think hamburger made with plant proteins from genetically modified (GM) yeast is unnatural. | 1=Strongly disagree  2=Disagree  3=Neither disagree or agree  4= Agree  5= Strongly agree |
|  | **H: COVID-19 Food Habits** |  |
| Prompt | **We will now ask you some questions about COVID-19.** | **Note to programmers: Ensure that Qualtrics coding matches the response scale, since it sometimes defaults to 0-4.** |
| H10  purchase | Compared to before the COVID-19 pandemic, would you say you now purchase more, less, or the same amount of red meat? | 1=A lot less  2=A little less  3=About the same  4=A little more  5=A lot more |
| H30  purchase_why | [display question only for those who replied less/a little less to H20]  [page break]  You said you now purchase less red meat than before the COVID-19 pandemic. Which of the following are reasons why you are not purchasing as much red meat as before COVID-19? | [display as check all that apply, randomize the list, leaving “other” at the bottom]  1=Red meat is too expensive  2=Red meat is sometimes out of stock at the store.  3=Red meat is perishable.  4=I have concerns about getting coronavirus from red meat  5=I have concerns about worker conditions in meat processing plants  5=Other, please describe |
|  | **D: Demographics** |  |
|  | **We will now ask you some questions about yourself.** |  |
| D10  age | How old are you? Enter your age in years. | [numeric free response, restricted to 18-99] |
| D15  Williams Institute  gender | How do you describe your gender identity? | 1=Male  2=Female  3=Non-binary  4=Prefer to self-describe:[free text] |
| D20  Hispanic ethnicity  ethnicity | Are you of Hispanic, Latino or Spanish origin? | 1=No, not of Hispanic, Latino, or Spanish origin  2=Yes, Mexican, Mexican American, Chicano  3=Yes, Cuban  4=Yes, another Hispanic, Latino, or Spanish origin (Enter Country of Origin) |
| D30  Race  race | What is your race? (check all that apply) | 1=White  2=Black or African American  3=American Indian or Alaska Native  4=Asian  5=Pacific Islander  6=Race not listed (please specify) |
| D40  Education  education | What is the highest level of education you have completed? | 1=Less than high school or U.S. high school equivalent (GED)  2=High school diploma or U.S. high school equivalent (GED)  3=Associate or technical degree  4=4-year college degree  5=Graduate degree (Master’s, PhD, or professional degree) |
| D50  Household Size  hhld_size | How many people are in your household, including you? | # of people [restricted to 1-20] |
| D60  Children in Household  hhld_child | How many children (ages 0-18) currently live in your household? | ____ [restricted to 0-15] |
| D70  Household income  income | Which of the following categories best describes your total household income in the last 12 months? | 1=Less than $10,000  2=$10,000 to $14,999  3=$15,000 to $24,999  4=$25,000 to $34,999  5=$35,000 to $49,999  6=$50,000 to $74,999  7=$75,000 to $99,999  8=$100,000 to $149,999  9=$150,000 to $199,999  10=$200,000 or more |
| D80  SNAP  snap | The next question is about SNAP, the Supplemental Nutrition Assistance Program, also known as the Food Stamp Program.  In the last **12 months**, did you or anyone in your household get SNAP or Food Stamps, even if only for one month? | 1=Yes  0=No |
| D90  Political affiliation  politics | Do you consider yourself to be: | 1=Liberal  2=Moderate  3=Conservative  [randomize order of items] |
|  | **Say how much you agree or disagree with the statements below.** |  |
| D100  Climate change  Benjamin et al (2016)  climate_occur | Climate change is occurring now. | 1 = Strongly disagree  2 = Somewhat disagree  3 = Neither agree nor disagree  4 = Somewhat agree  5 = Strongly agree |
| D110  Climate change  Benjamin et al (2016)  climate_human | The main cause of climate change is human activities. | 1 = Strongly disagree  2 = Somewhat disagree  3 = Neither agree nor disagree  4 = Somewhat agree  5 = Strongly agree |
|  | **Closure** |  |
| E10  Closure  closure | [page break] Anything you want to tell us about the study? Please leave your comments below. | [free text] |
| Ineligible statement | [DISPLAY IF INELIGIBLE]  Unfortunately, you are ineligible for this survey. Thank you for your time. |  |

Appendix C: CONSORT 2010 Checklist

| Section/Topic | Item No | Checklist item | Reported on page No |
| --- | --- | --- | --- |
| Title and abstract | | | |
|  | 1a | Identification as a randomised trial in the title | 1 |
|  | 1b | Structured summary of trial design, methods, results, and conclusions | 2-3 |
| Introduction | | | |
| Background and objectives | 2a | Scientific background and explanation of rationale | 3-5 |
|  | 2b | Specific objectives or hypotheses | 5 |
| Methods | | | |
| Trial design | 3a | Description of trial design (such as parallel, factorial) including allocation ratio | 8 |
|  | 3b | Important changes to methods after trial commencement (such as eligibility criteria), with reasons | N/A |
| Participants | 4a | Eligibility criteria for participants | 6 |
|  | 4b | Settings and locations where the data were collected | 6 |
| Interventions | 5 | The interventions for each group with sufficient details to allow replication, including how and when they were actually administered | 8-9 |
| Outcomes | 6a | Completely defined pre-specified primary and secondary outcome measures, including how and when they were assessed | 9-11 |
|  | 6b | Any changes to trial outcomes after the trial commenced, with reasons | N/A |
| Sample size | 7a | How sample size was determined | 6 |
|  | 7b | When applicable, explanation of any interim analyses and stopping guidelines | N/A |
| Randomisation: |  |  |  |
| Sequence generation | 8a | Method used to generate the random allocation sequence | 8 |
|  | 8b | Type of randomisation; details of any restriction (such as blocking and block size) | 8 |
| Allocation concealment mechanism | 9 | Mechanism used to implement the random allocation sequence (such as sequentially numbered containers), describing any steps taken to conceal the sequence until interventions were assigned | 9 |
| Implementation | 10 | Who generated the random allocation sequence, who enrolled participants, and who assigned participants to interventions | 8 |
| Blinding | 11a | If done, who was blinded after assignment to interventions (for example, participants, care providers, those assessing outcomes) and how | N/A |
|  | 11b | If relevant, description of the similarity of interventions | 9 |
| Statistical methods | 12a | Statistical methods used to compare groups for primary and secondary outcomes | 11-12 |
|  | 12b | Methods for additional analyses, such as subgroup analyses and adjusted analyses | 12 |
| Results | | | |
| Participant flow (a diagram is strongly recommended) | 13a | For each group, the numbers of participants who were randomly assigned, received intended treatment, and were analysed for the primary outcome | 6 |
|  | 13b | For each group, losses and exclusions after randomisation, together with reasons | 6 |
| Recruitment | 14a | Dates defining the periods of recruitment and follow-up | 6 |
|  | 14b | Why the trial ended or was stopped | N/A |
| Baseline data | 15 | A table showing baseline demographic and clinical characteristics for each group | Table 1 |
| Numbers analysed | 16 | For each group, number of participants (denominator) included in each analysis and whether the analysis was by original assigned groups | 16-17 |
| Outcomes and estimation | 17a | For each primary and secondary outcome, results for each group, and the estimated effect size and its precision (such as 95% confidence interval) | 13-17 |
|  | 17b | For binary outcomes, presentation of both absolute and relative effect sizes is recommended | N/A |
| Ancillary analyses | 18 | Results of any other analyses performed, including subgroup analyses and adjusted analyses, distinguishing pre-specified from exploratory | 13-14 |
| Harms | 19 | All important harms or unintended effects in each group | N/A |
| Discussion | | | |
| Limitations | 20 | Trial limitations, addressing sources of potential bias, imprecision, and, if relevant, multiplicity of analyses | 21-22 |
| Generalisability | 21 | Generalisability (external validity, applicability) of the trial findings | 22 |
| Interpretation | 22 | Interpretation consistent with results, balancing benefits and harms, and considering other relevant evidence | 17-21 |
| Other information | | |  |
| Registration | 23 | Registration number and name of trial registry | 12 |
| Protocol | 24 | Where the full trial protocol can be accessed, if available | N/A |
| Funding | 25 | Sources of funding and other support (such as supply of drugs), role of funders | 23 |
